# Gelatinous Barium for Roentgenology

**Published:** 1918-05

**Authors:** 


**Gelatinous Barium for Roentgenology.** Belot and Frauday, Bull. de l'Acad. de Med., Jan. 16, 1917, describe a new chemic form of barium of a creamy consistence, more agreeable to take, less liable to sedimentation and susceptible of incorporation with solid food, liquids or by itself. It is also claimed to be cheaper than the sulphate. The method of preparation is not described.

